# Scarce Evidence of Heterosis for Growth Traits in Peruvian Guinea Pigs

**DOI:** 10.3390/ani13172738

**Published:** 2023-08-28

**Authors:** José Isaí Cedano-Castro, Maria Wurzinger, Gustavo Gutiérrez, Ronald Jiménez, Amparo Elena Huamán Cristóbal, Johann Sölkner

**Affiliations:** 1 Facultad de Zootecnia, Universidad Nacional Agraria La Molina, Lima 150114, Peru; joiscecas@gmail.com (J.I.C.-C.);; 2 Programa de Estudio de Medicina Veterinaria y Zootecnia, Facultad de Ciencias Agrarias, Universidad Privada Antenor Orrego, Trujillo 13008, Peru; 3 Institute of Livestock Sciences, Department of Sustainable Agricultural Systems, BOKU—University of National Resources and Life Sciences, 1180 Vienna, Austria; 4 Instituto Veterinario de Investigaciones Tropicales y de Altura, Universidad Nacional Mayor de San Marcos, San Borja 15021, Peru

**Keywords:** crossbreeding, reciprocal crosses, productive traits

## Abstract

**Simple Summary:**

A method to improve meat production at guinea pig farms is by applying crossbreeding schemes. Our study evaluates the application of a two-way crossbreeding scheme using four genetic lines (two paternal and two maternal lines) to estimate the heterosis for productive traits. Positive heterosis effects in both types of crosses were found only for birth weight: 3.7% for paternal crosses and 12.7% for maternal crosses. However, the heterosis was not observed for weight at 10 days of age, weaning, or at 60 days of age (slaughter age). Based on this, applying a two-way crossbreeding scheme of paternal and maternal guinea pig lines for meat production would not be recommended for our population. On the other hand, assessing the three- or four-way crossbreeding schemes could be interesting.

**Abstract:**

This study aimed to estimate the heterosis for productive traits in a two-way crossbreeding scheme. Four guinea pig lines were originally selected for the following traits: line P1 for the growth rate, P2 for the partial feed conversion rate, M1 for the growth rate of the litter at 10 days of age, and M2 for the litter size at birth. The comparison included 176 purebreds (P1: 46, P2: 43, M1: 54 and M2: 33) and 150 crosses (P1P2: 42, P2P1: 38, M1M2: 11 and M2M1: 59); body weights at birth, 10 days, weaning and 60 days of age were analyzed. A linear fixed-effect model was used, and heterosis was estimated as the difference between the average performance of the crossbred and pure-line animals. The pure line comparisons showed that P2 was lower than P1 for weight at 10 days and weaning weight, while all other comparisons between the paternal and maternal pure lines were not significant. The results indicated significant positive heterosis effects for both types of crosses, but only for birth weight: 3.7% for paternal crosses and 12.7% for maternal crosses. The heterosis estimates were mostly positive but not significant for all other traits. A reason for the low levels of heterosis could be that the lines are not very genetically differentiated. These results suggest that applying a two-way crossbreeding scheme within paternal and maternal guinea pig lines for meat production is not recommended due to the absence of heterosis for growth traits.

## 1. Introduction

In Peru, guinea pig production is part of the Andean people’s life and culture. However, the high quality and nutritional value of guinea pig meat have transformed guinea pig production from a subsistence activity into a profitable business [1]. Guinea pig meat is considered as a healthy meat due to its high protein level (19%) of a high biological value, and it also has important minerals (1.2%) such as calcium, phosphorus, potassium, iron, and sodium and a low fat level (1.6%) [2].

During the last few decades, the guinea pig population has nearly tripled (from 6,884,938 to 19,725,802 animals between 1990 and 2019) [3]. This rising population has allowed farmers to increase the amount of guinea pig meat offered on local and international markets. Annually, Peru produces around 17 tons of guinea pig meat and exports around 10 tons of guinea pig meat to countries such as the United States of America, Japan, Italy, Canada, etc. [2]. In Peru, guinea pigs are sold alive or slaughtered, mainly at local markets or directly from farms, and their meat is only consumed on birthdays, anniversaries, or at fairs [4]. The high demand for guinea pig meat for special events causes the price of this meat to always be higher than that of other meats, such as chicken, pork, or beef [5,6].

Nowadays, in Peru, there are different breeds and genetic lines used for meat production, such as the Peru, Inti, Andina, and Kuri lines, developed by the INIA (Instituto Nacional de Innovación Agraria) [7,8,9]. The P1, P2, M1, and M2 lines were developed by the IVITA (Instituto Veterinario de Investigaciones Tropicales y de Altura) [10], the Cieneguilla breed developed by the UNALM (Universidad Nacional Agraria La Molina) [11], etc. Some guinea pig breeds or lines were established using the Peru, Inti, and Andina breeds as founder animals. The Kuri breed was established through a crossbreeding scheme using the three abovementioned breeds, and the P1 and M2 lines were developed using the Peru and Andina breeds, respectively [9,10].

All of these breeds enable farmers to increase their incomes. However, each breed or genetic line is managed under different production systems; for example, guinea pigs developed by the INIA or UNALM are reared under a traditional system, while the animals developed by IVITA are reared under a crossbreeding system [10]. The traditional system of guinea pig production implicates the eventual purchase of purebred males and the crossing of these males with females at the farms [4] versus the crossbreeding system promoted by the IVITA implicates the continual purchase of crossed males (F1 males) and females (F1 females) and only uses these animals for producing animals for slaughter at the farms [10].

Nowadays, the meat production of guinea pigs in Peru is carried out using breeds or genetic lines with improved growth traits. Breeds such as Peru, Inti, and Kuri are widely promoted in Peru because they reach the typical slaughter weight of 1000 g at around eight weeks of age. Moreover, their reproductive and fitness traits, such as the average litter size and high viability up to weaning, are highly remarkable in these breeds [1,9]. However, each region in Peru has developed regional genetic lines such as Mantaro, Saño [12], Inka, Chota [13], P1, P2, M1, M2 [10], etc. These lines are adaped to the climate conditions of the regions where they were originally developed. This offers an advantage in every step of the guinea pig production system in contrast to other breeds, as farmers can produce meat more efficiently.

Crossbreeding schemes for livestock are used for meat production. Two- and three-way crossbreeding schemes are used in beef, pork, rabbit, chicken, and broiler production systems [14]. These crosses are used to take advantage of heterosis and combine the desirable traits of two or three different breeds or genetic lines, thus producing terminal crossbred animals with stable and prominent performance [15]. Two-way crossbreeding schemes are commonly used in beef production systems [16]. However, three-way crossbreeding schemes (two maternal and one paternal or terminal breed) are mainly used in pig and rabbit production [14,17,18]. This strategy allows for an advantage in terms of the large litter sizes of the maternal lines and the productive traits of the terminal breed [14,18,19], as well as heterosis effects. 

Crossbred females (F1) are important, as they have larger litter sizes, better milk production, and better maternal abilities [14,19]. Furthermore, in four-way crossbreeding programs, F1 males are also produced by crossbreeding two different paternal breeds or lines. These F1 crossbreds show improved reproductive traits, such as sperm quality, motility, and viability. Moreover, both F1 females and males show higher fitness versus purebreds [14].

Therefore, the Instituto Veterinario de Investigaciones Tropicales y de Altura (IVITA) proposed a four-line crossbreeding scheme for the meat production of guinea pigs. Between 2007 and 2009, IVITA established two paternal (P1 and P2) and two maternal (M1 and M2) genetic lines. The P1 and P2 lines were selected for their high growth rate and low partial feed conversion rate [20], whereas M1 and M2 were selected for their high litter growth rate at 10 days of age and litter size at birth, respectively [10]. 

The four genetic lines were phenotypically similar to the main commercial breeds (Peru, Andina, and Inti) with regard to body shape, type of hair, and ear size [10,21]. The IVITA produces F1 males and females through the crossing of paternal and maternal lines, respectively. The IVITA provides both F1 males and females to farmers, which serve as a parent generation for the guinea pigs intended for meat consumption [10].

Therefore, this study aimed to estimate heterosis for growth traits in a two-way crossbreeding scheme for producing paternal and maternal types of F1 animals.

## 2. Materials and Methods

### 2.1. Location and Types of Animals

This research was carried out at the Instituto Veterinario de Investigaciones Tropicales y de Altura (IVITA) research center, located 3320 m above sea level in the El Mantaro district, Huancayo Province, Peru. This research center developed four guinea pig lines: two paternal (P1 and P2) and two maternal (M1 and M2). Line P1 is based on the genetic line “Peru”, and line M2 is based on the genetic line “Andina”, purchased from a commercial farm in Lima, Peru. Both are two well-established breeds that were developed by the INIA [7,8]. These breeds were established using animals from several Peruvian regions and selected to increase the animals’ weight gain at the time of slaughter (8 weeks of age) (Peru breed) and the litter size at weaning (Andina breed) [13]. On the other hand, P2 and M1 are developed by the IVITA and were established using local animals purchased from the neighboring farmers in Mantaro Valley. Following the development of the four guinea pig lines, the IVITA established specific selection criteria such as a growth rate for P1, partial feed conversion rate for P2, growth rate of the litter at 10 days of age for M1, and litter size at birth for M2. The four genetic lines are maintained as closed populations and are used in a four-way crossbreeding scheme established by the IVITA [10]. At the time of the study, lines P1, P2, M1, and M2 had 22, 25, 16, and 17 generations under the selection regime, respectively.

### 2.2. Study Design and Data Collection

Eight different crosses were performed, and four traits were recorded. Table 1 and Table 2 show different types of crosses and the number of traits recorded for the paternal and maternal lines, respectively.

The crosses were performed between September 2021 and September 2022. In total, 176 pure mates (P1: 46, P2: 43, M1: 54, and M2: 33) and 150 crosses (P1P2: 42, P2P1: 38, M1M2: 11, and M2M1: 59) were included in the analysis. M1M2 has a lower number of records due to its high mortality at birth. 

### 2.3. Statistical Analyses 

The statistical analyses were performed separately for each group of crosses (paternal and maternal). The effect of the type of cross was estimated through two different statistical linear models. Thus, for birth weight, weight at 10 days of age, and weaning weight, the following statistical model was used:

(1) y=μ+Type cross+Sex+Period+Litter size at birth+body condition+ε

For weight at 60 days of age, the statistic model used was:

(2) y=μ+Type cross+Sex+Period+Litter size at weaning+ε

where 

*y* = observations 

μ = overall mean

*Type cross* = type of mate or cross performed (4 levels, according to each group)

*Sex* = two levels (male and female)

*Period* = related to the season of the year, and was created using the birthday of animals (two levels, dry: between May and October and rainy: between November and April)

*Litter size at birth* = number of pups born in total (alive + dead)

*Litter size at weaning* = number of animals at weaning

*Body condition* of the mothers = 5 levels, where 1 is extremely thin, and 5 is extremely fat [22]

ε: environmental error associated with the observation.

Heterosis was estimated as the difference between the average of crossbred and pure mates:

(3) $$h_{AB}=\left({(\bar{X}}_{AB}+{\bar{X}}_{BA}\right)/2)-\left({(\bar{X}}_{AA}+{\bar{X}}_{BB}\right)/2)$$

The effect of the crossbred type was estimated through an analysis of variance, and the significance of the heterosis was estimated using orthogonal contrast, coding as −0.5 for pure lines and 0.5 for crossbred lines, according to the type of cross aforementioned in Table 1 and Table 2. These analyses were performed using RStudio software [23].

## 3. Results

### 3.1. Means of Productive Traits According to the Type of Crossbred

Table 3 and Table 4 show each trait’s mean and standard error according to the crossbred type. 

The results for P2 were lower than those of P1 for weight at 10 days of age and weaning weight. However, P2P1 reached higher means for birth weight than P2. However, all paternal crosses were similar for weight at 60 days of age (*p*-value > 0.05).

On the other hand, the performance for all the maternal crosses was similar for weight at 10 days of age, at weaning, and at 60 days of age (*p*-value > 0.05). However, the results for M1M2 were higher than those for the other types of crosses.

### 3.2. Heterosis

Table 5 shows heterosis estimated in grams and the percentage for the growth traits of the paternal and maternal crosses. Only the birth weight achieved significant heterosis.

## 4. Discussion

Crossbreeding schemes form part of several animal production systems and supply farmers with stable and good-performing animals [14,15]. However, this strategy has not been widely applied to local species such as guinea pigs, despite its advantages in terms of heterosis and combining traits expected for meat production, as described in a four-way crossbreeding scheme for this species [10]. 

The results for birth weight in seven of the eight experimental groups are in the range between 113 and 153 g as reported for other guinea pig genetic lines [12,24,25,26,27]. However, the M1M2 maternal crossbred achieved higher values (182 g) than the other crosses. These results suggest that the pure maternal lines give birth to smaller pups than the M1M2 crossbreed, due to the positive effects of crossing maternal lines. These results are also reflected in the significant heterosis effect of the maternal lines (Table 5).

The weight at 10 days of age is a trait that has scarcely been reported. Our results show that P1 reached higher values for this trait than P2 (*p*-value < 0.05). Moreover, both maternal lines, as well as the crosses, were similar weights at 10 days of age. The means estimated for weaning weight followed the trend of weight at 10 days of age in the paternal and maternal lines. Furthermore, some of the experimental groups (P1, M1, and M2M1) reached higher values for weaning weight than the range between 248 and 291 g reported in the literature [12,27]. It is important to take into account that we did not detect differences within the maternal crosses due to the low number of records.

The age at slaughter reported in the literature for guinea pigs is that at 56 days of age, with a target weight of 1000 g [1,26]. However, the means reported for weight at 56 days of age range between 614 and 1041 g [12,27,28]. This study shows the means for weight at 60 days of age, which range between 737 and 788 g in the paternal crossbreds and between 751 and 792 g in the maternal crossbreds, which are in the abovementioned range for weight at 56 days of age. One potential explanation for why the crosses did not reach the target weight might be the feeding regime. All these animals were fed with forage and a mix of grains as a supplement, which could have limited their growth. 

Heterosis in guinea pigs is rarely studied and reported in the literature. There are two studies where commercial guinea pig breeds were used. In one study, a comparison of crosses of the Peru × Andina and Peru × Inti breeds was carried out, and heterosis was estimated. This study found a low percentage of heterosis for weight at 85 days of age (1.3 and 1.5%), daily weight gain (−3.0 and 3.9%), and the feed conversion rate (7.4 and 7.8%) in each cross, the Peru × Andina and Peru × Inti, respectively [29]. In a Colombian study, two synthetic guinea pig lines (5/8P_3/8N and 5/8N_3/8P) were developed through a crossbreeding scheme between Colombian (N) and Peruvian (P) guinea pig breeds. This study reported low-positive as well as negative percentages of heterosis for the same traits that we evaluated in our study, such as birth weight (1.40% and −6.29%), weaning weight (0.83% and −7.82%), and weight at 56 days of age (−10.97% and −16.14%), in 5/8P_3/8N and 5/8N_3/8P genetic lines, respectively [30].

In a crossbreeding experiment of Peruvian and Bolivian guinea pigs estimating components of heterosis, based on a complex, multi-generation crossbreeding experiment, no dominant components of heterosis were found for growth traits or for reproductive traits. The results were conflicting, depending on the model of heterosis applied [31]. Our study shows significant positive heterosis for birth weight in both types of crosses, while low-positive and negative values of heterosis for all the other weight traits were observed, as also observed by other authors [29,30,31]. Low heterosis was also found in crossbred rabbits and pigs for productive traits under two-way crossbreeding schemes [32,33]. This supports the idea that traits with moderate heritability, such as growth traits, are not highly improved by crossing [14,34].

Our results could also be explained by the close relationship between the pure lines involved. Very small Fst values (0.0013) were reported between the Peru and Andina breeds [35], which were used to establish the P1 and M2 lines [10]. Moreover, the Fst values between the Peru and Andina breeds did not improve, and the Peru (commonly wrongly called “native”) guinea pigs were also small (Fst 0.0182 and 0.0155, respectively) [35]. This report suggests that all guinea pig breeds or genetic lines are closely related. This low genetic differentiation could be the key reason for the low heterosis estimated in this study [14,34]. 

## 5. Conclusions

The results of this study suggest low levels of heterosis for growth traits in first-generation crosses (F1) of guinea pigs. Yet, it would be interesting to evaluate a three-way crossbreeding scheme using the F1 maternal crossbreed as a dam line and P1 or P2 line as a terminal sire. Thus, to take advantage of the combined desirable traits of these three different guinea pig lines, heterosis for growth, litter size, and other economically important traits should also be estimated for these types of crosses.

## Figures and Tables

**Table 1:** Traits and number of records in paternal line crosses (P1, P2, P1P2, P2P1).

Type of Cross	Birth Weight	Weight at 10 Days	Weaning Weight *	Weight at 60 Days
P1	143	136	122	92
P2	123	121	117	96
P1P2	139	133	133	101
P2P1	124	117	122	93
TOTAL	529	507	494	382

* Age at weaning: 15 days of age.

**Table 2:** Traits and number of records in maternal line crosses (M1, M2, M1M2, M2M1).

Type of Cross	Birth Weight	Weight at 10 Days	Weaning Weight *	Weight at 60 Days
M1	158	126	125	88
M2	78	72	69	53
M1M2	11	11	11	4
M2M1	60	59	56	36
TOTAL	307	268	261	181

* Age at weaning: 15 days of age.

**Table 3:** Means and standard errors (in parenthesis) for growth traits in paternal lines crosses.

Type of Cross	Birth Weight (g)	Weight at 10 Days (g)	Weaning Weight (g)	Weight at 60 Days (g)
P1	140 (4.2) ^ab^	250 (7.7) ^a^	314 (9.7) ^a^	788 (18.8)
P2	127 (4.3) ^b^	211 (7.9) ^b^	261 (9.9) ^b^	745 (18.7)
P1P2	135 (4.1) ^ab^	232 (7.5) ^ab^	294(9.3) ^ab^	744 (17.8)
P2P1	142 (3.9) ^a^	235 (7.3) ^ab^	295 (9.9) ^ab^	737 (16.1)

^a,b^ Values in the same column with different superscripts are statistically different (*p* < 0.05).

**Table 4:** Means and standard errors (in parenthesis) for growth traits in maternal lines crosses.

Type of Cross	Birth Weight (g)	Weight at 10 Days (g)	Weaning Weight (g)	Weight at 60 Days (g)
M1	149 (4.1) ^a^	249 (6.5)	314 (7.3)	783 (15.4)
M2	150 (4.0) ^a^	247 (6.0)	299 (7.2)	756 (19.0)
M1M2	182 (9.0) ^b^	247 (13.2)	292 (15.8)	792 (59.8)
M2M1	155 (4.8) ^a^	261 (7.2)	316 (8.7)	751 (25.8)

^a,b^ Values in the same column with different superscripts are statistically different (*p* < 0.05).

**Table 5:** Heterosis for growth traits in paternal and maternal line crosses.

Trait	Heterosis					
	Paternal Crosses			Maternal Crosses		
	g.	%	Sig.	g.	%	Sig.
Birth weight	5.4 (2.2)	3.7	*	18.8 (4.9)	12.7	*
Weight at 10 days	3.1 (4.1)	1.3	ns	5.7 (7.2)	2.4	ns
Weaning weight	7.0 (5.1)	2.4	ns	−3.1 (8.7)	−0.8	ns
Weight at 60 days	−26.0 (14.6)	−3.4	ns	1.6 (34.9)	0.3	ns

g.: grams, %: percentage based on the average of the pure mate; Sig., significance of heterosis, * = significant (*p*-value < 0.05), ns = not significant.

## Data Availability

Not applicable.
